# Examining the Relationship Between Extreme Temperature, Microclimate Indicators, and Gestational Diabetes Mellitus in Pregnant Women Living in Southern California

**DOI:** 10.1097/EE9.0000000000000252

**Published:** 2023-05-31

**Authors:** Anais Teyton, Yi Sun, John Molitor, Jiu-Chiuan Chen, David Sacks, Chantal Avila, Vicki Chiu, Jeff Slezak, Darios Getahun, Jun Wu, Tarik Benmarhnia

**Affiliations:** a Herbert Wertheim School of Public Health and Human Longevity Science, University of California San Diego, La Jolla, California; b School of Public Health, San Diego State University, La Jolla, California; c Department of Environmental and Occupational Health, Program in Public Health, University of California, Irvine, California; d College of Public Health and Human Sciences, Oregon State University, Corvallis, Oregon; e Departments of Population & Public Health Sciences and Neurology, University of Southern California, Los Angeles, California; f Department of Research & Evaluation, Kaiser Permanente Southern California, Pasadena, California; g Department of Obstetrics and Gynecology, University of Southern California, Keck School of Medicine, Los Angeles, California; h Department of Health Systems Science, Kaiser Permanente Bernard J. Tyson School of Medicine, Pasadena, California; i Scripps Institution of Oceanography, University of California San Diego, La Jolla, California

**Keywords:** Extreme temperature, Effect modification, Gestational diabetes mellitus, Microclimate

## Abstract

Few studies have assessed extreme temperatures’ impact on gestational diabetes mellitus (GDM). We examined the relation between GDM risk with weekly exposure to extreme high and low temperatures during the first 24 weeks of gestation and assessed potential effect modification by microclimate indicators.

**Methods:**

We utilized 2008–2018 data for pregnant women from Kaiser Permanente Southern California electronic health records. GDM screening occurred between 24 and 28 gestational weeks for most women using the Carpenter-Coustan criteria or the International Association of Diabetes and Pregnancy Study Groups criteria. Daily maximum, minimum, and mean temperature data were linked to participants’ residential address. We utilized distributed lag models, which assessed the lag from the first to the corresponding week, with logistic regression models to examine the exposure-lag-response associations between the 12 weekly extreme temperature exposures and GDM risk. We used the relative risk due to interaction (RERI) to estimate the additive modification of microclimate indicators on the relation between extreme temperature and GDM risk.

**Results:**

GDM risks increased with extreme low temperature during gestational weeks 20–-24 and with extreme high temperature at weeks 11–16. Microclimate indicators modified the influence of extreme temperatures on GDM risk. For example, there were positive RERIs for high-temperature extremes and less greenness, and a negative RERI for low-temperature extremes and increased impervious surface percentage.

**Discussion:**

Susceptibility windows to extreme temperatures during pregnancy were observed. Modifiable microclimate indicators were identified that may attenuate temperature exposures during these windows, which could in turn reduce the health burden from GDM.

**What this study adds**

This study contributes to the literature by providing a new understanding of the role of extreme temperatures on maternal health outcomes. It addresses the impacts of both extreme high and low-temperature exposures on GDM risk at the weekly level as well as the modification of extreme temperatures and GDM risk by microclimate indicators. By identifying windows of susceptibility during pregnancy to extreme temperatures and examining modifiable microclimate indicators, improved strategies aimed at the use of preventative behaviors or policy implementation that influence these microclimate indicators may be used to attenuate temperature exposures that could, in turn, reduce GDM risk.

## Introduction

A range of maternal complications can occur during pregnancy, including anemia, hypertension, mental health issues, thromboembolic disorders, and gestational diabetes mellitus (GDM), among others, and these complications can cause increased health risks to the mother and fetus.^1–5^ GDM, a form of glucose intolerance that has an onset during pregnancy, affects approximately 5%–10% of pregnancies within the United States, and its incidence has been rising over time.^6–8^

Certain characteristics can predispose individuals to an increased GDM risk, such as obesity, older childbearing age, family history of diabetes, parity, race, ethnicity, and previous history of GDM.^9,10^ Furthermore, environmental factors can contribute to an increased risk of GDM. This includes characteristics, such as increased air pollution,^11–13^ extreme temperature exposure,^14^ and louder road traffic noise.^15^ Of these environmental exposures, although many studies have assessed air pollution impacts on GDM,^12,13,16–19^ fewer studies have focused on the role of temperature on GDM risk. Previous studies have focused on exposure to high average temperatures and seasonality on GDM.^14,20–27^ These studies demonstrated that GDM risk was higher during the summer months than in the winter months, and GDM risk increased with increasing temperatures. However, most of these studies utilized average temperature and examined this relation at the monthly- or seasonal level. To our knowledge, only one study has assessed exposure to both extreme high and low temperatures and GDM risk.^28^ Additionally, this relation has rarely been investigated at more refined temporal scales, such as the weekly level during pregnancy. Investigating this relation would allow for potential extreme temperature thresholds and windows of susceptibility to these temperatures during pregnancy to be identified. This can provide insight for early alert systems and warn pregnant women to utilize protective behaviors during these temperature thresholds or specific gestational weeks.

Furthermore, some studies have assessed microclimate indicator impacts, including green space presence and the normalized difference vegetation index (NDVI), on GDM risk, which are exposures that may modify the impact of extreme temperatures.^11,22,29^ These studies’ findings suggested that increased residential proximity to greenness and greenness exposure led to a decreased GDM risk. However, other microclimate indicators, including tree canopy, imperviousness, and land surface temperature, have not yet been considered in studies assessing environmental exposures and GDM risk, despite their known influence on variations in local temperatures.^30–33^ Additionally, while the literature has focused on how urban landscape may influence variations in micro-heat islands, it may also drive variations in extreme low temperatures and associated health impacts.^34^ If these microclimate characteristics influence the relation between extreme temperatures and GDM risk, then urban planning policies may be implemented, such as planting more trees and/or reducing impervious surfaces by using higher albedo manmade surfaces, as a means of attenuating extreme temperatures, which would, in turn, reduce the risk of GDM. Identifying these potentially modifiable microclimate indicators is especially important in the context of climate change, as both high- and low-extreme temperatures may become more severe.^25^ The assessment of microclimate indicator modification and the identification of extreme temperature thresholds would allow for improved individual and community-based preventive measures to be implemented, which could assuage extreme temperatures and indirectly minimize GDM risk.

In this study, we assessed GDM risk with weekly exposure to extreme air temperature (maximum, minimum, and average) during the first 24 weeks of gestation, and we examined whether microclimate indicators including NDVI, tree canopy, impervious surfaces, and land surface temperature, among others, modify this relation. This analysis used data from electronic health records for pregnancies delivered in the Kaiser Permanente Southern California (KPSC) healthcare system. This large population-based cohort of pregnant women presents a unique opportunity to study these relationships.^35,36^

## Methods

### Study population

This retrospective cohort study included women who gave birth between January 1, 2008, and December 31, 2018, at KPSC facilities, which include 15 hospitals and 234 medical offices across Southern California. Women without residential address data (n = 680), those with multiple births (n = 7,454), those who were not KPSC members during the study period, or those with pregnancy lasting <20 or >47 gestational weeks (n = 8,912) at the time of miscarriage or delivery, respectively, were excluded from this study. Pregnancies with preexisting diabetes (n = 5,518) and those with missing laboratory test results on GDM status (n = 30,355) were also excluded. In total, 395,927 pregnancies were included in this analysis. All residential addresses were geocoded with the Texas A&M, NAACCR, Automated Geospatial Geocoding Interface Environment Geocoder.^37^ The gestational week was determined by the date of onset of the last menstrual period and corroborated by early pregnancy ultrasonography. Information on demographic characteristics, residential history, medical records, birth history, and individual lifestyle was extracted from KPSC electronic health records (EHRs). Further details of the cohort have been previously described.^36^ This study was approved by the Institutional Review Board of KPSC and the University of California, Irvine with a waiver of informed consent.

### GDM outcome

GDM diagnosis was based on KPSC laboratory tests that were routinely performed between 24 and 28 gestational weeks, except for women at higher risk for GDM who are screened earlier. Similarly to Sun et al.^36^ two criteria for GDM testing were used: the Carpenter-Coustan criteria (a 1-hour 50-g glucose challenge test >200 mg/dL or two or more abnormal values for 3-hour 100-g oral glucose tolerance test [OGTT], using the cutoff values fasting ≥95, 1 hour ≥180, 2 hour ≥155, 3 hour ≥140 mg/dl); or the International Association of Diabetes and Pregnancy Study Groups criteria (one or more abnormal values for a 2-hour 75-g OGTT, using the cutoff values fasting ≥ 92, 1hour ≥ 180, 2hour ≥ 153 mg/dl).^36,38^

### Ambient temperature exposure

Historical daily temperature data from January 1, 2007 to December 31, 2018 were derived from the spatiotemporal, high-resolution Gridded Surface Meteorological dataset.^39^ This dataset provides daily surface fields of maximum temperature (T_max_) and minimum temperature (T_min_) at a 4-km spatial resolution covering the contiguous United States. The gridded meteorological data were validated against automated weather stations across the complex topography of the western United States,^39^ and they are available via the Climatology Lab—gridMET product.

For creating individual-level exposure to ambient air temperature, gridded daily temperature estimates were spatiotemporally linked to each woman based on the geocoded residential addresses during pregnancy. Because approximately 44% of the population moved during pregnancy in this population, information on residential changes (address, start date, and end date) were also extracted from KPSC EHRs. The ambient air temperature exposures were collected based on a given individual’s accurate residential address for that particular day, thus incorporating if they had moved during pregnancy. We then calculated T_max_, T_min_, and T_mean_ ([T_max_+ T_min_]/2) in every gestational week by averaging these variables on the first day of the corresponding week and the following 6 days. The time-varying exposure based on the exact diagnosis date was not accounted for, because GDM screening and diagnosis is generally performed between 24 and 28 gestational weeks. In our study, approximately 80% of GDM cases were diagnosed after the 20th gestational week, and only some high-risk women (12%) may have been screened early in their pregnancy (during the first trimester). Thus, we explored the effect of weekly-specific maternal temperature exposure on GDM risk by accounting for all past (lagged) exposures from 1 to 24 gestational weeks as performed in previous studies.^19,28^

In this study, T_max_, T_min_, and T_mean_ were used to explore the effect of extreme temperature exposure. We focused on the 1st and 3rd percentiles of each temperature variable as our extreme low-temperature exposures, while the 97th and 99th percentiles were defined as extreme high-temperature exposures. These heat wave and cold spell definitions were based on the temperature distribution of each participant’s neighborhood. For each threshold of extreme temperatures, mothers were considered exposed if they experienced (at least) one extreme high- or low-temperature event during a given gestational week. We thus considered 12 weekly temperature exposures (3 temperature variables and 4 percentiles).

### Modification by microclimate indicators

We additionally explored the potential effect modification of microclimate indicators on the relation between extreme temperature exposures and GDM risk. Such microclimate indicators are critical to study, as they may influence extreme temperatures. This information could be particularly useful to tailor interventions to minimize the impact of extreme temperature exposures on GDM risk. We aggregated these indicators through satellite remote sensing products such as Moderate Resolution Imaging Spectroradiometer and Landsat, which provide high spatial and temporal resolution images of these indicators. We primarily focused on the 2011 percentage of the tree canopy, the 2013 percentage of developed impervious surfaces, the 2018 land surface temperature, and the 2018 average NDVI. These have a spatial resolution of 30 m, 30 m, 70 m, and 250 m, respectively. Additional indicators were explored including evapotranspiration canopy, evapotranspiration soil, evaporative stress index, landcover classification, and water use efficiency, as these factors may provide further insight regarding the implementation of actionable policies, such as replacing impermeable surfaces with more permeable surfaces or planting trees and other types of vegetation. We hypothesized that effect modification existed by increasing impervious surfaces, evaporative stress index, and more developed land cover, which would exacerbate the relation between increasing temperatures and GDM risk, and by increasing tree canopy percentage, NDVI, evapotranspiration canopy, evapotranspiration soil, and water use efficiency, which would attenuate the relation of interest. Raster files were aggregated from Google Earth Engine, and 200-meter buffers (as a priori buffer size to consider residential exposure) were created surrounding the residential addresses to find the average microclimate indicators for each participant.

### Covariates

Covariate data were extracted from KPSC EHRs. Pregnancy-related covariates and potential confounders were selected *a priori* based on the existing literature,^28,40^ which included maternal age at delivery; race/ethnicity (African American, Asian, Hispanic, non-Hispanic white, and others including Pacific Islanders, Native American/Alaskan, and multiple race/ethnicities specified); maternal educational level (≤ 8th grade, 9th grade to high school, college <4 years, and college ≥4 years); median household income at block group level in 2013 (categorized as quartiles)^41^; pre-pregnancy body mass index (BMI) (underweight: <18.5 kg/m^2^, normal: 18.5–24.9 kg/m^2^, overweight: 25.0–29.9 kg/m^2^, and obese: ≥30.0 kg/m^2^); exposure to active smoking (never smoker, ever smoker, and smoking during pregnancy); passive (i.e., secondhand) smoking (yes or no); parity (1, 2, 3, and ≥4); infant sex (male and female); year of infant birth (2008–2018); and the season of conception (warm season: May–October, and cool season: November–April). Previous studies have included the season of conception covariate with four categories,^28,42^ while others have utilized this as a binary variable as we did.^43^

### Statistical analysis

Descriptive statistics of population characteristics were assessed for the total study population and stratified by GDM and non-GDM status. For our main analyses, we examined the exposure-lag-response associations between each of our 12 weekly extreme temperature exposures and GDM risk by implementing distributed lag models (DLMs) coupled with logistic regression as performed previously.^44,45^ Such models consider current exposure at a given time t, past exposure before time t (as our main approach, we used an inverse weighting approach with weights being calculated based on time to a week t to give more weight to weeks right before a week t), and potential interactions between past (i.e., week_1_–week_t-1_) and current (i.e., week_t_) exposures. The lag range was defined as completed gestational weeks from the first week to the corresponding week. Odds ratios (ORs) and 95% confidence intervals (CIs) were calculated for the probability of GDM occurrence for each of the 12 temperature exposures. Main analyses adjusted for maternal age, race/ethnicity, education, median household income, pre-pregnancy BMI, active or passive smoking during pregnancy, insurance type, season of conception, and year of birth.

As a sensitivity analysis, we added both parity and the sex of the infant as potential confounders that were additionally adjusted for in the DLM models, given that these covariates have been adjusted for in previous studies. We then explored alternative functional forms for the potential lagged effects by distributed lag nonlinear models (DLNMs) with logistic regression.^46^ The median temperature of each temperature variable was used as the reference temperature.^28^ DLNMs were applied to calculate ORs and 95% CIs of GDM risk at the 3rd and 97th percentiles of each temperature variable relative to median temperature (as models did not converge for temperature definitions based on 1st and 99th percentiles). Natural cubic splines were used to define the lag-response effect, and we used three degrees of freedom for all temperature indicators to explore such alternative nonlinear relationships.

In the effect modification analysis, we used the relative risk due to interaction (RERI) to estimate the additive interactive effect of extreme temperature exposure and microclimate indicators on the risk of GDM. We first calculated temperature exposures by averaging the daily temperature measurements during the first 24 gestational weeks, and we considered the same 12 binary temperature exposures as in our main analyses. These temperature exposure definitions were context-specific, where the distribution of each participant’s neighborhood was utilized. The RERIs were calculated per one-unit increase in each microclimate variable (some indicators were reversed to facilitate the interpretation). RERI = $OR_{11}-OR_{10}-OR_{01}+1$, where $OR_{xz}=Pr\left(Y=1|X=x,Z=z\right)$ is the risk of the outcome Y when the first exposure X is value x (e.g., extreme temperature exposure, in the context of this study) and the second exposure Z is value z (e.g., a particular microclimate indicator).^47,48^ All analyses were performed in SAS version 9.4 (SAS Institute, Inc., Cary, NC) and R (version 4.0.5).

## Results

Table 1 provides the descriptive statistics for T_mean,_ T_min_, and T_max_ across the first 24 weeks of gestation. Temperature averages for T_mean,_ T_min_, and T_max_ were found to be 18.7ºC (SD: 3.2)_,_ 12.3ºC (SD: 3.2), and 25.0ºC (SD: 3.7), respectively. The Supplementary Material (http://links.lww.com/EE/A221) includes summary statistics of characteristics for the full population and stratified by the GDM outcome (Table S1; http://links.lww.com/EE/A221). Large differences were observed for GDM by maternal race and ethnicity, where more Asian and Hispanic mothers had GDM, and by prepregnancy BMI, where more obese mothers had GDM.

**Table 1.** Summary statistics of weekly temperature indicators (unit: ºC) during the 1st to 24th gestational weeks among participants.

Temperature	Mean	SD	Min	1st	3rd	50th	97th	99th	Max
T_mean_	18.7	3.2	0.4	11.3	13.1	18.5	24.1	25.1	28.2
T_min_	12.3	3.2	−7.0	3.9	6.0	12.3	17.5	18.3	34.8
T_max_	25.0	3.7	5.8	18.1	19.4	24.5	32.4	33.7	43.7

Units are degree Celsius for temperature indicators.

SD indicates standard deviation; T_max_, daily maximum temperature; T_mean_, daily mean temperature; T_min_, daily minimum temperature.

**Figure 1.**
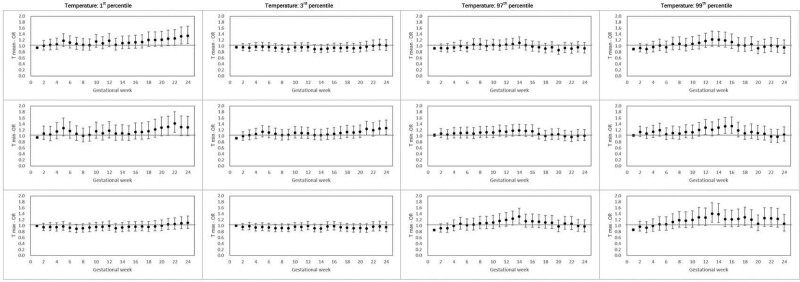
DLM models depicting the relation between T_mean_, T_min_, and T_max_ and GDM for the 1st, 3rd, 97th, and 99th percentiles of temperature across the first 24 weeks of gestation. DLM indicates distributed lag models; T_max_, daily maximum temperature; T_mean_, daily mean temperature; T_min_, daily minimum temperature.

The results from the main DLM models are depicted in Figure 1, which shows the relation between the 12 extreme temperature exposures and GDM across the first 24 gestational weeks. For the 1st percentile of temperature, while variation across weeks can be noted, ORs tend to become higher (i.e., an increased GDM risk) in the second half of the gestational weeks for T_mean_ and T_min_, where weeks 20–24 show a significant 22%–35% increase in GDM risk. In comparison, weaker but increased GDM risks were observed for T_min_ for the 3rd percentile of temperature, where weeks 21–24 have positive ORs with more precise CIs (i.e., narrow 95% CIs that exclude 1.0), while the T_mean_ estimates tend to remain consistently but imprecisely negative. Similarly, for both the 1st and 3rd percentiles (extreme low temperatures), the T_max_ estimates tend to be predominantly negative and imprecise. For the 97th percentile (extreme high temperatures), T_max_ had a bell-shaped curve for its estimates across gestational weeks, with negative or null estimates for the first trimester and late second trimester and positive estimates for weeks 13–14. Although T_mean_ and T_min_ followed a similar pattern for the 97th percentile estimates, they were close to null and insignificant. The shape of the 99th temperature percentile over the gestational weeks was similar to that of the 97th percentile, although more pronounced. Both T_min_ and T_max_ have more precise, positive ORs from gestational weeks 11–16, indicating approximately 21%–41% higher risks of GDM.

Sensitivity analyses show that parity and infant sex did not change the results substantially. Figure S1; http://links.lww.com/EE/A221 shows results considering DLNM with 3 degrees of freedom. Across T_mean_, T_min_, and T_max_ for the 3rd and 97th percentiles of temperature, we found a similar pattern of negative or null estimates during the first few gestational weeks, followed by positive values for weeks 6–18, negative values from weeks 18 to 22, and an upward trend for the remaining 22–24 weeks.

**Figure 2.**
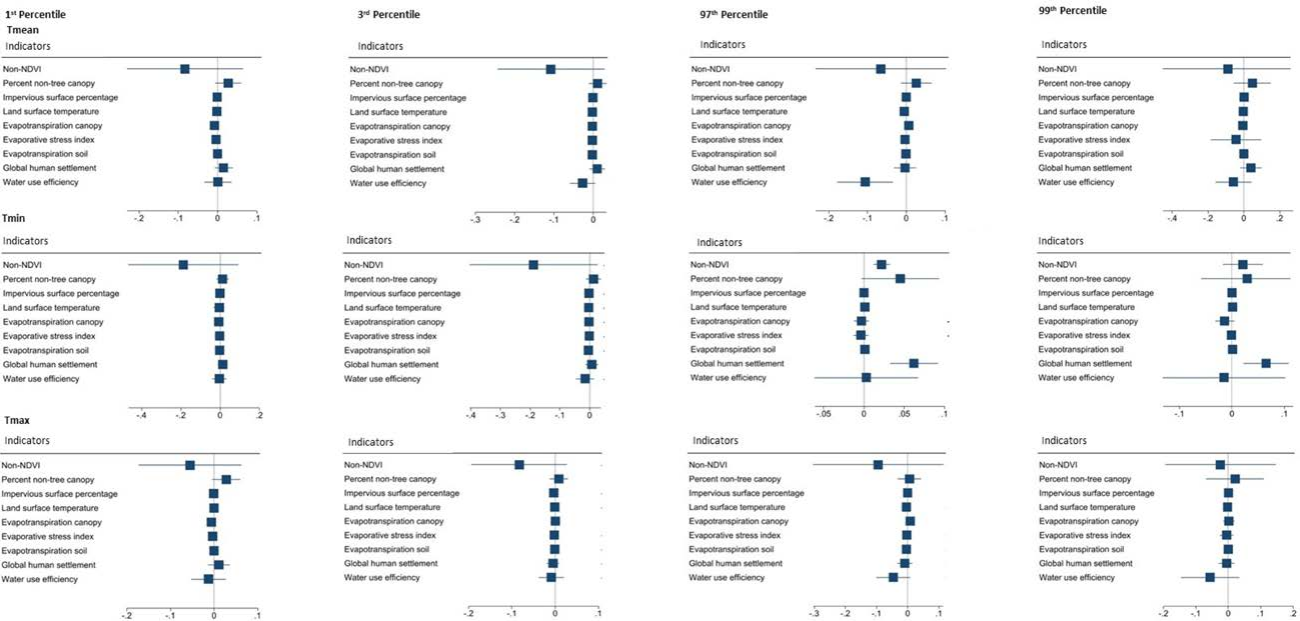
Forest plots depicting the RERIs between extreme temperature and microclimate indicators. The first row provides T_mean_, the center row provides T_min_, and the last row provides T_max_. From left to right, the columns are as follows: 1^st^, 3^rd^, 97^th^, and 99^th^ percentiles. RERI indicates relative risk due to interaction; T_max_, daily maximum temperature; T_mean_, daily mean temperature; T_min_, daily minimum temperature.

Finally, Figure 2 provides forest plots for the effect modification (on the additive scale) by the microclimate indicators (Table S2; http://links.lww.com/EE/A221 for the RERI estimate and 95% CI values). At low-temperature extremes, a negative RERI was identified between T_max_ and increased impervious surface percentage, meaning the combined effects were lower than expected. At high-temperature extremes, we found a negative RERI between T_mean_ and higher water use efficiency (i.e., lower than expected joint effects), while positive RERIs were observed between T_min_ and non-NDVI, increased land surface temperature, and global human settlement, T_max_ and higher evapotranspiration canopy, and T_mean_ and higher evapotranspiration canopy (i.e., synergistic effect, or higher than expected joint effects).

## Discussion

In this study, we assessed both the weekly exposure-lag-response associations between 12 extreme high- and low-temperature exposures and GDM risk and the RERIs to assess the additive effect modification by microclimate indicators. We identified an increased GDM risk between weeks 20 and 24 of gestation for extreme low-temperature exposures. In contrast, we observed an increased GDM risk for gestational weeks 11–16 for extreme high-temperature exposures. We also found that microclimate indicators modified the influence of extreme temperatures on GDM risk.

Although a clear trend was not identified across exposure definitions regarding GDM risk, windows of susceptibility during certain gestational weeks were observed during extreme high- and low temperatures. Many studies have assessed this relation later in pregnancy and have identified an increased GDM risk with increasing average temperature as well as the presence of seasonal variation, where GDM risk and prevalence are higher in the warmer seasons than in the colder seasons^23,25,27^; however, most of these studies have not assessed the impact of high temporal-resolution, the weekly time-varying temperature on GDM risk. Vasileiou et al^20^ and Katsarou et al^49^ investigated the monthly variation in the relation between temperature and GDM risk, and they identified that GDM diagnosis was higher during the summer months. Molina-Vega et al^21^ additionally assessed this monthly-level relation and seasonal variation, and they identified a higher GDM diagnosis prevalence during warmer seasons and increased temperatures in the 2–4 weeks before oral glucose tolerance testing. To our knowledge, only one other study has addressed this relation at the weekly level and examined longer-term exposures during pregnancy; Zhang et al^28^ identified that extreme low-temperature exposures increased GDM risk between gestational weeks 14 and 17, while extreme high-temperature exposures increased GDM risk between weeks 21 and 24. Thus, these susceptibility windows differed but overlapped between the study by Zhang et al^28^ and our study. This may be explained by differences in climate conditions (e.g., lower temperature distributions in our study than that of Zhang et al^28^, possible differences in humidity levels, etc.) or population composition (e.g., maternal age, prepregnancy BMI, household income, tobacco smoking, fetal gender, and parity), for example. However, increased risk from both extreme high- and low-temperature was identified during the second trimester of pregnancy in both studies. Identifying these susceptibility windows has implications for maternal health and interventions to minimize extreme temperature exposures during these windows.

Furthermore, effect modification by microclimate indicators on the relationship between temperature and GDM risk was observed. Although studies have explored the direct effects of some microclimate indicators on GDM risk as exposures of interest (e.g., Liao et al^11^, Preston et al^22^, and Qu et al^29^, identifying that increased greenness could lead to a decrease in GDM risk), few have assessed modification by these indicators on the associations of extreme temperatures with GDM. Other microclimate indicators, including impervious surfaces, tree canopy, and land surface temperature (as a direct measure of local variation in temperatures), have neither been considered as exposures of interest nor as effect modifiers. Investigating these possible effect modifications is critical, as these microclimate indicators are modifiable determinants of GDM, meaning that if these microclimate indicators were acted on, this may assuage extreme temperatures and in turn potentially reduce the risk of GDM. We found effect modification by lower NDVI, higher land surface temperature, lower water use efficiency, increased global human settlement, and higher evapotranspiration canopy, which aggravate the relation between extreme high temperature and GDM risk, while a higher developed impervious surface percentage attenuates the harmful influence of extreme low temperature on GDM risk. Future work may wish to simulate the intervention effects of these modifiable microclimate indicators on GDM risk in entire populations or in susceptible sub-populations, which could have critical policy implications.

Previous studies have hypothesized biological mechanisms that may explain the influence of extreme temperature on the development of GDM. At higher temperatures, insulin sensitivity diminishes, leading to increased insulin resistance.^23^ Furthermore, venous plasma glucose levels tend to increase due to core temperature-associated blood flow redistribution.^23,25,50^ In contrast, brown adipose tissue glucose uptake increases in cold temperatures, which in turn improves insulin sensitivity.^25^ Although brown adipose tissue activation at lower temperatures may improve glucose metabolism through increased insulin sensitivity, other mechanisms may explain glucose level changes and subsequent GDM development as a result of colder temperatures.^25^ One explanation includes vitamin D_3_ deficiency, which is related to sunlight exposure that may be minimized during periods of colder temperatures—or periods of high temperature during which individuals minimize their time outside—as well as dietary intake, and this can lead to glucose metabolism issues and thus a higher risk of GDM.^23,25^ However, mixed results have been identified related to the plausibility of the influence of sunlight exposure and vitamin D deficiency on GDM risk.^25,50,51^ Previous studies have also suggested that extreme high- and low-temperature exposure may lead to the activation of an inflammatory response, and this may in turn influence GDM risk.^28,51^ Future studies may wish to further examine the mechanisms between extreme temperatures and GDM risk.

The main strengths of this study include the large, diverse KPSC pregnancy cohort; comprehensive and high-quality clinical data, especially for GDM diagnosis based on laboratory glucose tolerance tests rather than diagnostic codes or recall information; the consideration of both extreme low- and high-temperature exposure; and accurate exposure estimates accounting for residential history during pregnancy. However, certain limitations exist. It is possible for extreme temperature exposure and microclimate indicators to be misclassified, as these were defined using geocoded residential addresses. Although we incorporated changes in the residential address during pregnancy, we did not utilize mobility data that could take into consideration individual time-activity patterns and dynamic environmental exposures. Thus, it is possible that these participants were exposed to extreme temperatures and microclimate indicators in areas other than their residential homes (e.g., workplace). This use of stationary outdoor temperature exposures would likely contribute to nondifferential misclassification, and this could lead to bias towards the null if the population was exposed to more extreme temperatures outside of their place of residence. Furthermore, regarding the microclimate indicators, these were averaged across a given year within the study period (e.g., 2013 for the percentage of impervious surfaces, 2018 for NDVI) but not averaged across the entire study period of January 1, 2008, through December 31, 2018. Indoor temperatures were also not accounted for, making it not possible to differentiate between locations that the participants were in that utilized or did not utilize certain adaptation strategies (ex. air conditioning). In California, 75% of the population owns either room or central air conditioning,^52^ and, more specifically within Southern California, 44% and 56% of the population in San Diego and Los Angeles, respectively, have central air conditioning in their household.^53^ Furthermore, there may be behavioral variability of these participants that was not considered, especially during periods of extreme high or low temperatures. This bias resulting from the lack of information related to adaptive strategies or protective behaviors would likely be differential misclassification, given that some but perhaps not all of the population utilize these strategies or behaviors, potentially contributing to bias away from the null. We also did not account for time-varying exposure based on the onset time of GDM. This is due to the uncertainty related to whether the diagnosis date reflects the true onset time of GDM. For most pregnant women, screening is performed between 24 and 28 gestational weeks; however, some high-risk women were screened earlier during the first trimester of their pregnancy. Thus, we could not assess the exact onset of GDM given these screening criteria. Moreover, it is possible that patients identified as having GDM by one set of criteria may not have been identified by the other. We also focused on binary temperature exposures as opposed to continuous temperature exposures. Although defining cutoffs for binary exposures may be arbitrary and information may be lost,^54^ these binary definitions for extreme low and extreme high-temperature definitions may be helpful to inform and design warning systems that are often based on a threshold to trigger a set of preventive actions. Such thresholds may be used to target populations that are more at risk, such as pregnant women, to ultimately reduce GDM risk.^55^ The women included in this study had access to this insurance, thereby potentially being healthier relative to the target population (Southern California pregnant mothers). Nevertheless, the KPSC population, in general, is representative of the southern California population.^56^ These limitations serve as possible future research avenues: incorporating personal behaviors such as utilizing air conditioning, heating systems, or cooling centers, and assessing this relation between extreme temperature and GDM risk among other populations or climatic zones.

## Conclusions

This study addressed the impact of extreme high and low temperatures on GDM risk and the modification of this relation by microclimate indicators. Specific windows of susceptibility to both extreme high and low temperatures were identified during the second trimester of pregnancy, allowing for preventative behaviors to be used during this time to limit these exposures. Furthermore, effect modification was identified by non-NDVI, impervious surface percentage, land surface temperature, water use efficiency, global human settlement, and evapotranspiration canopy, highlighting the possibility of incorporating actionable interventions that may modify these microclimate indicators, which could assuage extreme temperatures and in turn may reduce GDM risk. Identifying these susceptibility windows and modifiable microclimate indicators are particularly important, as these extreme temperatures may become more frequent and severe due to climate change.

## Conflict of interest statement

The authors declare that they have no conflicts of interest with regard to the content of this report.

## Supplementary Material


