# Supplementary material for: Examining the Relationship Between Extreme Temperature, Microclimate Indicators, and Gestational Diabetes Mellitus in Pregnant Women Living in Southern California
Source: Environ Epidemiol. 2023 May 31;7(3):e252. doi: 10.1097/EE9.0000000000000252 (PMC10256373; doi:10.1097/EE9.0000000000000252)
Supplement: Supplementary file 1 [file ee9-7-e252-s001.docx]

**Supplementary Material**

**Table S1.** Selected population characteristics by Gestational diabetes (GDM) groups, 2008-2018.

| **Characteristics** | **GDM**  **n = 42,970** | **Non-GDM**  **n = 352,957** | **Total births**  **n = 395,927** |
| --- | --- | --- | --- |
| Maternal age, years, mean (SD) | 32.6 (5.3) | 30.0 (5.7) | 30.3 (5.7) |
| Maternal race/ethnicity, n (%) | | | |
| African American | 2273 (5.3) | 27776 (7.9) | 30049 (7.6) |
| Asian | 8833 (20.6) | 42775 (12.1) | 51608 (13.1) |
| Hispanic | 22549 (52.6) | 166627 (47.3) | 189176 (47.8) |
| Non-Hispanic white | 7445 (17.4) | 97702 (27.7) | 105147 (26.6) |
| Multiple/other | 1804 (4.2) | 17597 (5.0) | 19401 (4.9) |
| Maternal education, n (%) | | | |
| ≤ 8th grade | 778 (1.8) | 2974 (0.9) | 3752 (1.0) |
| 9th grade – high school | 12319 (29.2) | 104595 (30.2) | 116914 (30.1) |
| College (< 4 years) | 9594 (22.7) | 78448 (22.7) | 88042 (22.7) |
| College (4 years) | 14026 (33.2) | 111956 (32.4) | 125982 (32.4) |
| > College | 5485 (13.0) | 48078 (13.9) | 53563 (13.8) |
| Median household income at block group level in 2013, n (%) | | | |
| ≤ $43,973 | 11401 (26.6) | 87270 (24.8) | 98671 (25.0) |
| $43,973-$56,396 | 11292 (26.4) | 87382 (24.8) | 98674 (25.0) |
| $56,397-$72,032 | 10806 (25.2) | 87903 (25.0) | 98709 (25.0) |
| > $72,032 | 9360 (21.8) | 89261 (25.4) | 98621 (25.0) |
| Smoking, n (%) | | | |
| Never Smoker | 35967 (83.7) | 294903 (83.6) | 330870 (83.6) |
| Ever Smoker | 4992 (11.6) | 40189 (11.4) | 45181 (11.4) |
| Smoking during pregnancy | 2011 (5.1) | 17852 (4.7) | 19863 (5.0) |
| Passive smoker, n (%) | | | |
| Yes | 693 (1.6) | 7686 (2.2) | 8379 (2.1) |
| No | 42267 (98.4) | 345013 (97.8) | 387280 (97.9) |
| Insurance type, n (%) | | | |
| Medicaid | 3241 (7.6) | 32967 (9.5) | 36208 (9.3) |
| Other insurance type | 39240 (92.4) | 315331 (90.5) | 354571 (90.7) |
| Season of conception, n (%) | | | |
| Warm season | 20492 (47.7) | 174705 (49.5) | 195197 (48.5) |
| Cool season | 22478 (52.3) | 178252(50.5) | 200730 (51.5) |
| Pre-pregnancy BMI in categories, n (%) | | | |
| Underweight (<18.5 kg/m^2^) | 521 (1.2) | 9135 (2.6) | 9656 (2.5) |
| Normal (18.5-24.9 kg/m^2^) | 11610 (27.2) | 159565 (45.5) | 171175 (43.5) |
| Overweight (25.0-29.9 kg/m^2^) | 12635 (29.6) | 98469 (28.1) | 111104 (28.2) |
| Obese (≥ 30.0 kg/m^2^) | 17904 (42.3) | 83467 (23.8) | 101374 (25.8) |
| Parity | | | |
| 1 | 15797 (36.9) | 149115 (42.4) | 164912 (41.8) |
| 2 | 13458 (31.4) | 113370 (32.3) | 126828 (32.2) |
| 3 | 8031 (18.8) | 55412 (15.8) | 63443 (16.1) |
| ≥ 4 | 5532 (12.9) | 33605 (9.6) | 39137 (9.9) |
| Infant sex, n (%) | | | |
| Female | 20764 (48.3) | 172694(48.9) | 193458 (48.9) |
| Male | 22203 (51.7) | 180230 (51.1) | 202433 (51.1) |

SD, standard deviation; BMI, body mass index. The units are µg/m^3^ for PM_2.5_ and PM_10_, and parts per billion for NO_2_.

**Figure S1.** Maternal weekly-specific extreme high and low temperature defined by temperature indicators and the risk of GDM by distributed lag non-linear models incorporating logistic regression (with 3 degrees of freedom).


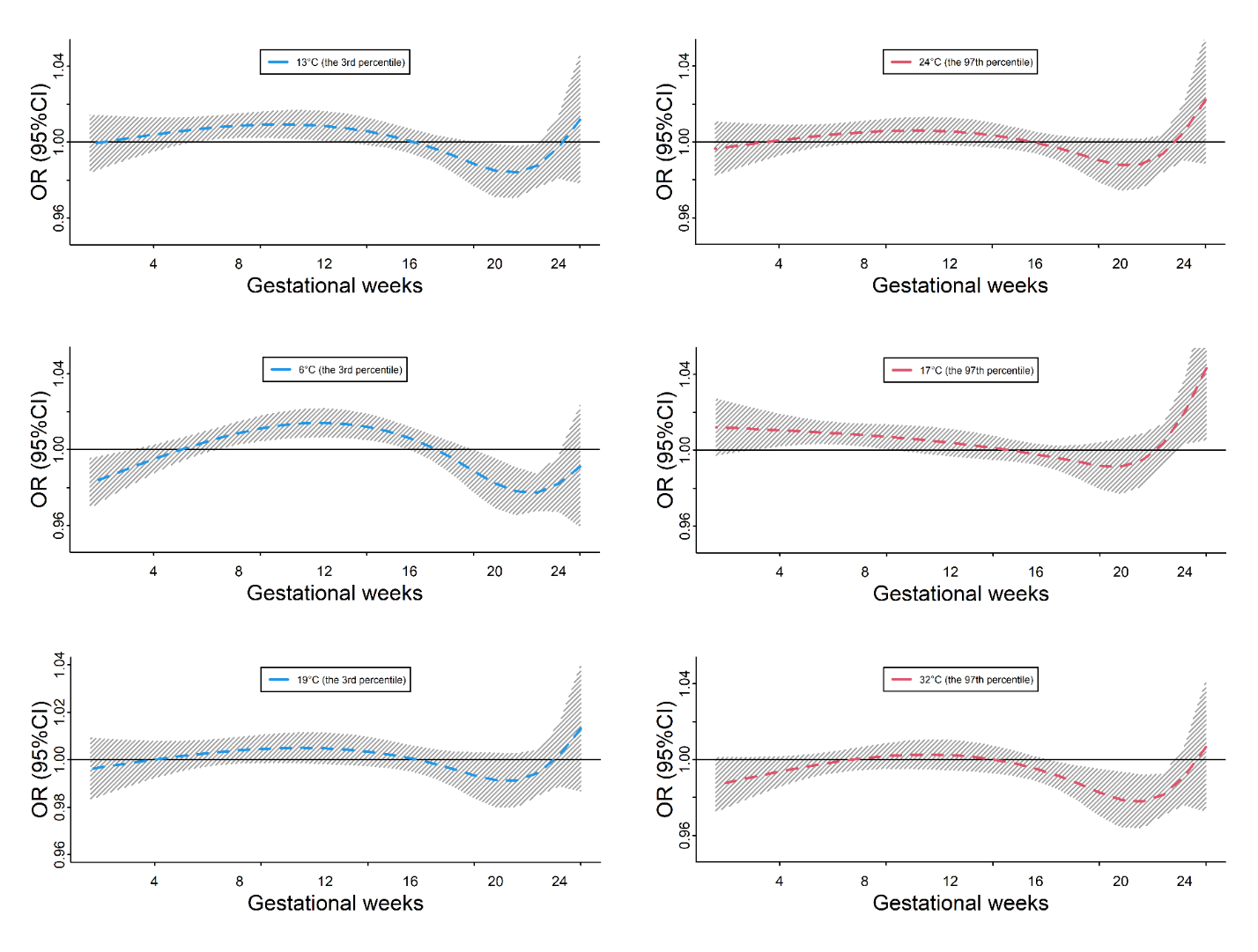


T_mean_, daily mean temperature; T_min_, daily minimum temperature; T_max_, daily maximum temperature; Units are degree Celsius for temperature indicators. The reference temperatures for T_mean_, T_min_, and T_max_ were 19℃, 12℃, and 25℃, respectively; All models adjusted for maternal age, race/ethnicity, education, household income, pre-pregnancy BMI, smoking during pregnancy, insurance type, season of conception and year of birth.

**Table S2.** Interaction between temperature and microclimate indicators. Results are provided for T_mean_, T_min_, and T_max_ for the 1^st^, 3^rd^, 97^th^, and 99^th^ percentile of temperature. RERI and 95% confidence intervals are included.

| Threshold Temperature | **1^st^ percentile** | **3^rd^ percentile** | **97^th^ percentile** | **99^th^ percentile** |
| --- | --- | --- | --- | --- |
| **Mean temperature** | | | | |
| Non-NDVI | -0.083 (-0.230, 0.064) | -0.108 (-0.243, 0.027) | -0.066 (-0.233, 0.102) | -0.089 (-0.441, 0.285) |
| Percent non-tree canopy | 0.027 (-0.005, 0.060) | 0.011 (-0.011, 0.033) | 0.026 (-0.013, 0.064) | 0.048 (-0.055, 0.151) |
| Impervious surface percentage | -0.001 (-0.011, 0.008) | -0.001 (-0.003, 0.002) | 0 (-0.003, 0.003) | 0.001 (-0.004, 0.006) |
| Land surface temperature | -0.002 (-0.011, 0.008) | -0.002 (-0.007, 0.004) | -0.005 (-0.009, 0) | -0.003 (-0.009, 0.003) |
| Evapotranspiration canopy | -0.008 (-0.015, 0) | -0.002 (-0.007, 0.003) | **0.007 (0.0003, 0.015)** | -0.006 (-0.025, 0.012) |
| Evaporative stress index | -0.004 (-0.014, 0.005) | -0.002 (-0.007, 0.003) | -0.003 (-0.012, 0.006) | -0.043 (-0.181, 0.096) |
| Evapotranspiration soil | 0 (-0.003, 0.003) | -0.002 (-0.006, 0.002) | -0.001 (-0.006, 0.004) | 0 (-0.008, 0.008) |
| Global human settlement | 0.015 (-0.008, 0.037) | 0.01 (-0.008, 0.029) | -0.003 (-0.032, 0.025) | 0.04 (-0.018, 0.098) |
| Water use efficiency | 0.001 (-0.033, 0.035) | -0.027 (-0.058, 0.005) | **-0.106 (-0.177, -0.035)** | -0.058 (-0.159, 0.042) |
| **Minimum temperature** | | | | |
| Non-NDVI | -0.187 (-0.467, 0.093) | -0.189 (-0.404, 0.026) | **0.022 (0.012, 0.032)** | 0.021 (-0.017, 0.059) |
| Percent non-tree canopy | 0.013 (-0.017, 0.042) | 0.013 (-0.011, 0.037) | 0.045 (-0.003, 0.093) | 0.029 (-0.058, 0.116) |
| Impervious surface percentage | -0.001 (-0.004, 0.002) | -0.002 (-0.005, 0) | 0 (-0.004, 0.004) | 0 (-0.006, 0.006) |
| Land surface temperature | -0.004 (-0.031, 0.022) | -0.003 (-0.018, 0.012) | **0.001 (0.0004, 0.002)** | 0.001 (-0.002, 0.004) |
| Evapotranspiration canopy | -0.006 (-0.013, 0.002) | -0.002 (-0.007, 0.003) | -0.003 (-0.012, 0.006) | -0.014 (-0.032, 0.003) |
| Evaporative stress index | -0.002 (-0.007, 0.003) | -0.001 (-0.003, 0.002) | -0.004 (-0.013, 0.005) | -0.001 (-0.01, 0.007) |
| Evapotranspiration soil | -0.002 (-0.007, 0.003) | -0.004 (-0.01, 0.002) | 0.001 (-0.003, 0.004) | 0.001 (-0.007, 0.008) |
| Global human settlement | 0.014 (-0.01, 0.037) | 0.007 (-0.014, 0.027) | **0.062 (0.032, 0.091)** | **0.065 (0.022, 0.108)** |
| Water use efficiency | -0.003 (-0.04, 0.034) | -0.015 (-0.045, 0.015) | 0.003 (-0.061, 0.067) | -0.015 (-0.131, 0.102) |
| **Maximum temperature** | | | | |
| Non-NDVI | -0.055 (-0.172, 0.062) | -0.083 (-0.193, 0.027) | -0.095 (-0.303, 0.113) | -0.024 (-0.194, 0.145) |
| Percent non-tree canopy | 0.028 (-0.003, 0.060) | 0.009 (-0.012, 0.029) | 0.006 (-0.031, 0.042) | 0.021 (-0.067, 0.109) |
| Impervious surface percentage | -0.001 (-0.004, 0.002) | **-0.003 (-0.005, -0.0001)** | 0 (-0.003, 0.003) | 0.001 (-0.004, 0.007) |
| Land surface temperature | 0 (-0.004, 0.004) | -0.001 (-0.003, 0.001) | -0.004 (-0.009, 0.0002) | -0.002 (-0.009, 0.006) |
| Evapotranspiration canopy | -0.006 (-0.013, 0.002) | 0.001 (-0.004, 0.005) | **0.008 (0.001, 0.015)** | 0.002 (-0.014, 0.018) |
| Evaporative stress index | -0.003 (-0.011, 0.004) | -0.002 (-0.007, 0.002) | -0.003 (-0.011, 0.005) | -0.005 (-0.026, 0.016) |
| Evapotranspiration soil | 0 (-0.002, 0.003) | -0.001 (-0.004, 0.002) | -0.004 (-0.012, 0.004) | 0 (-0.005, 0.006) |
| Global human settlement | 0.011 (-0.013, 0.035) | -0.005 (-0.018, 0.009) | -0.009 (-0.033, 0.015) | -0.005 (-0.028, 0.019) |
| Water use efficiency | -0.013 (-0.052, 0.026) | -0.009 (-0.038, 0.019) | -0.046 (-0.099, 0.006) | -0.056 (-0.145, 0.033) |
